# Effective communication in benefit-risk assessment: the role of visual tools

**DOI:** 10.1590/S2237-96222026v35e20251403.en

**Published:** 2026-06-24

**Authors:** Ana Carolina Nonato, Erica Aranha Suzumura, Bruna de Oliveira Ascef, Aline Frossard Ribeiro Bortoluzzi, Fernando Henrique de Albuquerque Maia, Sidney Marcel Domingues, Beate Jahn, Uwe Siebert, Patrícia Coelho de Soárez

**Affiliations:** 1 Universidade de São Paulo, Faculdade de Medicina, São Paulo, SP, Brazil Universidade de São Paulo Faculdade de Medicina São Paulo SP Brazil; 2 UMIT TIROL - University for Health Sciences and Technology, Institute of Public Health, Medical Decision Making and Health Technology Assessment, Hall in Tirol, Tirol, Austria UMIT TIROL - University for Health Sciences and Technology Institute of Public Health Medical Decision Making and Health Technology Assessment Hall in Tirol Tirol Austria; 3 ONCOTYROL - Center for Personalized Cancer Medicine, Division of Health Technology Assessment, Innsbruck, Tirol, Austria ONCOTYROL - Center for Personalized Cancer Medicine Division of Health Technology Assessment Innsbruck Tirol Austria; 4 Harvard T.H. Chan School of Public Health, Center for Health Decision Science and Departments of Epidemiology and Health Policy & Management, Boston, MA, United States Harvard T.H. Chan School of Public Health Center for Health Decision Science Departments of Epidemiology and Health Policy & Management Boston MA United States; 5 Harvard Medical School, Mass General Brigham Center for Health Technology Assessment, Boston, MA, Estados Unidos Harvard Medical School Mass General Brigham Center for Health Technology Assessment Boston MA Estados Unidos

Visual tools play a fundamental role in benefit-risk assessment, as they facilitate communication of complex information and enhance understanding between different stakeholders. This article presents the main types of tools for presenting benefit-risk assessments used in the context of health technology assessment and discusses important aspects for their selection and appropriate application.

## Main types and characteristics of visual tools

A scoping review ^1^ of methodological guidelines and publications that guided the conduct and reporting of benefit-risk assessments throughout the life cycle of health technologies identified, in 74 documents, the types of visual tools most frequently used. Effect and evidence tables were the most frequently used tools (n=49; 59.0%), followed by value trees (n=31; 41.0%), bar charts (n=28; 33.7%), scatter plots (n=28; 33.7%), line graphs (n=26; 31.3%) and area charts (n=24; 28.9%). These categories were not mutually exclusive, so it was common for the same publication to use more than one type of visual tool.

Among the various visual tools used in benefit-risk assessment, the value tree (Figure 1) and the effects table (Table 1) stand out for their structuring role in organizing criteria and consolidating quantitative evidence. Although not present in all assessments, these tools are frequently used as a basis for defining relevant outcomes and for transparent synthesis of information that underpins the benefit-risk judgment.

**Figure 1:**
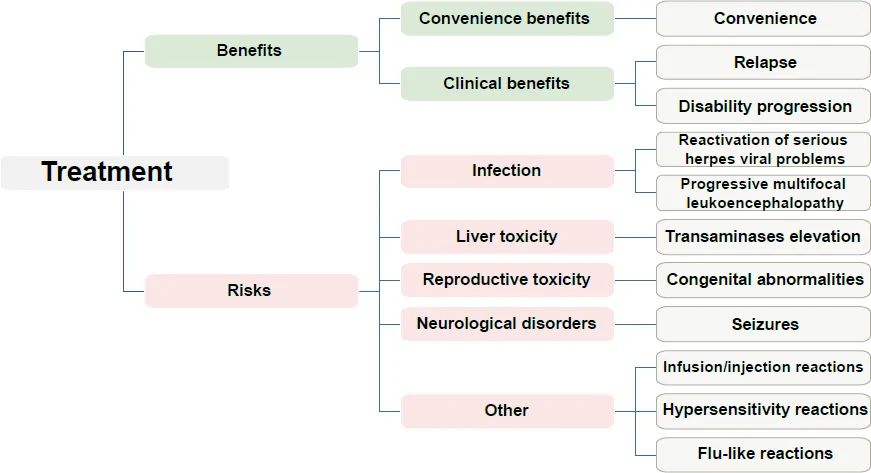
Outcomes selected to represent benefits and risks of using natalizumab versus comparator. Adapted from Nixon et al. ^7^

**Table 1:** Benefíts and risks associated with natalizumabe versus placebo, according to the outcomes assessed. Adapted from Nixon et al. ^7^

Result	Outcome	Natalizumabe Absolut risk per 1000 patients	Placebo Absolut risk per 1000 patients	Absolut risk difference per 1000 patients (95%CI)	
Benefits					
Convenience benefits	Convenience	-	-	--	
Clinical benefits	Relapse	280.00	450.00	-260.00 (-326.000; -195.00)	
	Disability progression	110.00	230.00	-120.00 (--)	
**Risks**					
Infection	Reactivation of serious herpes viral problems herpes	80.00	70.00	10.00 (-26.00; 45.00)	
	Progressive multifocal leukoencephalopathy	2.00	0.00	2.00 (--)	
Liver toxicity	Transaminases elevation	50.00	40.00	10.00 (-16.00; 38.00)	
Reproductive toxicity	Congenital abnormalities	-	-	-	--
Neurological disorders	Seizures	-	-	-	--
**Other**	Infusion/injection reactions	236.00	180.00	56.00 (6.00; 114.00)	
	Hypersensitivity reactions	90.00	40.00	50.00 (20.00; 82.00)	
	Flu-like reactions	399.00	400.00	-1.00 (-114.00; 114.00)	

**Legend:** green indicates the absolut risk difference of occurrence of the event that favors natalizumab (benefit or reduction of adverse events in relation to the comparator); pink indicates the absolut risk difference of occurrence of the event that favors natalizumab (increase in events or greater risk compared to placebo).

The value tree clearly and structurally represents the relationship between the benefits and risks considered in the benefit-risk assessment (Figure 1). Its development typically occurs in meetings of multidisciplinary teams responsible for the benefit-risk assessment, where it acts as a tool for discussion and recording of the most relevant elements of the evaluation process. The value tree focuses on the main events related to the benefit-risk assessment and does not aim to present an exhaustive list of events, in either the benefits or the risk dimension, but rather to highlight the most relevant benefits and risks that a decision-maker considers when choosing between different technologies ^2^.

The value tree can be used in any methodology involving multiple outcomes to facilitate understanding of the problem structure. Generally, a value tree is developed in the initial stages of analytical frameworks such as PrOACT-URL ^3^ or BRAT ^4^ and can also be used to guide the weighting process in quantitative methods, such as multi-criteria decision analysis ^5^. It is crucial that a value tree be presented together with information about the context in which it was created, as, on its own, it does not provide sufficient detail about the reasons for the inclusion or exclusion of certain criteria. In order to avoid misinterpretations or biases, it is essential to explicitly state the assumptions and ensure transparency in the selection of benefits and risks presented in a value tree, ideally incorporating clinical experience and patient perspectives. Furthermore, since a value tree does not provide quantitative data, such as metrics of association or effect, it can lead to misinterpretations if not properly assessed, as it does not indicate associations or measures of strength of evidence between the criteria presented ^5^^),(^^6^. It is also important to ensure that the events represented in a value tree correspond to the events identified in the clinical studies used as sources of evidence for the benefit-risk assessment.

Table 1, an effects table, presents an overview of the numerical values ​​of the clinical benefits and risks associated with a given technology. The data displayed in an effects table are purely descriptive, i.e., they do not incorporate any formal analysis of information on preference. In some cases, the data may clearly show that a health technology presents a favorable benefit-risk balance versus its comparator(s). When the data in an effects table do not indicate a clear advantage of any alternative technology, for example, when the technology of interest offers not only additional benefits but also additional risks, quantitative modeling for benefit-risk assessment with calculation of explicit trade-off measures, such as the incremental harm-benefit ratio, may be considered ^5^.

Beyond descriptive presentation, an effects table can integrate data from formal benefit-risk analyses, including assignment of weights to outcomes and explicit calculation of benefit-risk contributions. In this approach, quantitative evidence and preferences regarding outcomes are incorporated in a transparent and structured way, as illustrated in Table 2. The effects table is widely used in the PrOACT-URL analytical framework and in multi-criteria decision analysis ^6^^)-(^^8^. When preparing it, it is important to strike a balance between the amount of information presented and ease of reading, as excessively dense tables can increase the reader’s cognitive load and hinder extraction of the most relevant information.

**Table 2:** Absolute differences in the likelihood of occurrence of the event per 1000 patients and respective 95% confidence intervals (95% CI) for benefits and risks associated with natalizumab versus placebo, according to the outcomes assessed, weighted by the multi-criteria decision analysis method^a^. Adapted from de Nixon et al. ^7^

	Outcome	Natalizumabe Absolut risk per 1000 patients	Placebo Absolut risk per 1000 patients	Absolut risk difference per 1000 patients (95%CI)	Weight (%)	Benefit-risk
Benefits						
Convenience benefits	Convenience	--	--	--	1.00	-0.30
Clinical benefits	Relapse (number of relapses)	467.00	1460.00	-993.00 (-1267.00; -756.00)	8.00	3.97
	Disability progression	113.00	230.00	-117.00 (-124.00; -110.00)	5.00	0.59
**Risks**						
Infection	Reactivation of serious herpes viral problems	0.00	0.00	0.00 (-0.00; 6.30)	6.00	0.00
	Progressive multifocal leukoencephalopathy	1.51	0.00	1.51 (0.00; 3.00)	54.00	-0.08
Liver toxicity	Transaminases elevation	50.00	40.00	10.00 (-19.00; 36.00)	11.00	-0.11
Reproductive toxicity	Congenital abnormities	0.00	0.00	0.00 (-6.00; 3.00)	5.00	0.00
Neurological disorders	Seizures	5.00	5.00	0.00 (-2.00; 12.00)	5.00	0.00
**Other**	Infusion/injection reactions	236.00	0.00	236.00 (202.00; 269.00)	3.00	-0.71
	Hypersensitivity reactions	0.00	0.00	0.00 (-6.00; 3.00)	1.00	0.00
	Flu-like reactions	399.00	400.00	-1.00 (-90.00; 86.00)	1.00	0.00
**Total**					100.00	3.36

**Note:** ^a^This table presents the structured benefit-risk assessment, comparing natalizumab versus placebo using the PrOACT-URL framework and the multi-criteria decision analysis method. Outcomes are grouped into benefit and risk domains (in the first columns). The absolut risk of occurrence of events per 1,000 patients for natalizumab and placebo are presented, followed by the absolute risk difference with their respective 95% confidence intervals (95%CI). Each outcome received a relative weight defined in the multi-criteria decision analysis. The benefit-risk contribution of each criterion was calculated from the absolut risk difference weighted by its respective weight. The overall benefit-risk result is presented in the last column as the weighted sum of all criteria. Positive values indicate that the benefits of natalizumab outweigh the risks compared to placebo.

**Legend:** The color-coding of the likelihood difference column indicates the direction of the effect: green indicates outcomes that favor natalizumab; red indicates outcomes that favor the comparator.

The evidence table is similar to the table used to present certainty of evidence assessed according to the criteria proposed by the GRADE (Grading of Recommendations Assessment, Development and Evaluation) method ^9^. In benefit-risk assessment, an evidence table should include outcomes considered important or critical, whether benefits or risks, and the quality of the evidence should be assessed and presented, indicating the level of certainty in the effect estimates and the factors that influence it, such as risk of bias, inconsistency or imprecision ^10^^),(^^11^.

Bar charts can be used to compare the efficacy and safety of different treatments, illustrate the incidence of adverse events, or demonstrate the distribution of results in different patient groups. It is important to emphasize that, when using bar charts in this context, care must be taken to ensure that the scale and proportions are accurately represented ^6^^)-(^^8^.

The dot plot is a simplified alternative to the bar chart and can display a variety of metrics, such as frequencies, likelihoods, proportions and other results by group. The main advantage of this tool is that it presents only crucial data points, eliminating unnecessary visual elements.

The forest plot is a more sophisticated variation of the dot plot, incorporating more robust statistical fundamentals, and is particularly useful for communicating summary metrics, such as differences between absolute risks and risk ratios, with their respective confidence intervals. This characteristic makes the forest plot especially valuable in communicating benefit-risk assessments, as it allows for a quick visual assessment of the magnitude and precision of the observed effects ^6^^)-(^^8^.

Line graphs communicate relationships and changes in metrics over a range of values, allowing visualization of trends, patterns and relationships between variables. They are particularly useful for highlighting dose-response relationships (e.g., frequency of adverse events in relation to drug dosage) or for illustrating variations in the effectiveness of a treatment over time ^6^^)-(^^8^.

Area graphs and volume charts can be used to visually compare prevalence of risks and benefits between alternative technologies. A significant limitation of these graphics is the human difficulty in accurately perceiving areas and volumes. Consequently, although these graphics can effectively illustrate the existence of differences in observed effects among different health technologies, it can be difficult to accurately determine the magnitude of these differences ^6^^)-(^^8^.

Pictograms, in turn, play an important role in conveying complex information in an accessible way to diverse audiences, such as regulators, patients and the general public, as they are more easily understood than other graphics. They can be used to visually represent the incidence of adverse events and benefits of treatments or other health technologies, with particular utility for communicating likelihoods and proportions ^6^^)-(^^8^.

## Selecting appropriate visual tools

Selection of the appropriate visual tool for benefit-risk assessment depends on several factors, such as the type of information to be communicated, the target audience and the stage of the analysis. Compatibility between the images and the target audience must be determined, as well as the main message to be communicated, considering the level of knowledge required to understand the graph or figure, and whether there is sufficient information for the intended message to be clearly communicated and understood ^6^^)-(^^8^.

In the assessment planning phase, using the value tree is recommended in order to visualize the hierarchical structure of the decision problem, in addition to preparing a table model to represent the data to be collected. The effects table should be completed during evidence collection and data preparation, highlighting the available and missing information. Use of risk scales or pictograms is suggested for presenting information to the general public ^6^^)-(^^8^.

In the quantitative analysis stage, stakeholder value preferences and benefit-risk magnitudes can be represented by bar charts, scatter plots, line graphs or forest plots. These visual representations promote accurate reading of points, local and global comparisons, and facilitate judgment between alternatives. Finally, in the exploration phase, when results are checked for robustness due to changes in parameters and statistical uncertainty, using a distribution plot, a tornado diagram or a boundary analysis plot is recommended to represent the distribution or uncertainty of a metric ^6^^)-(^^8^.

Figure 2 systematizes the objectives and main types of visual tools to be used. Additional examples of these tools, developed by the PROTECT BR project ^6^, are available for consultation at: https://imi-protect-eu.cc.ic.ac.uk/visualisations.html.

**Figure 2:**
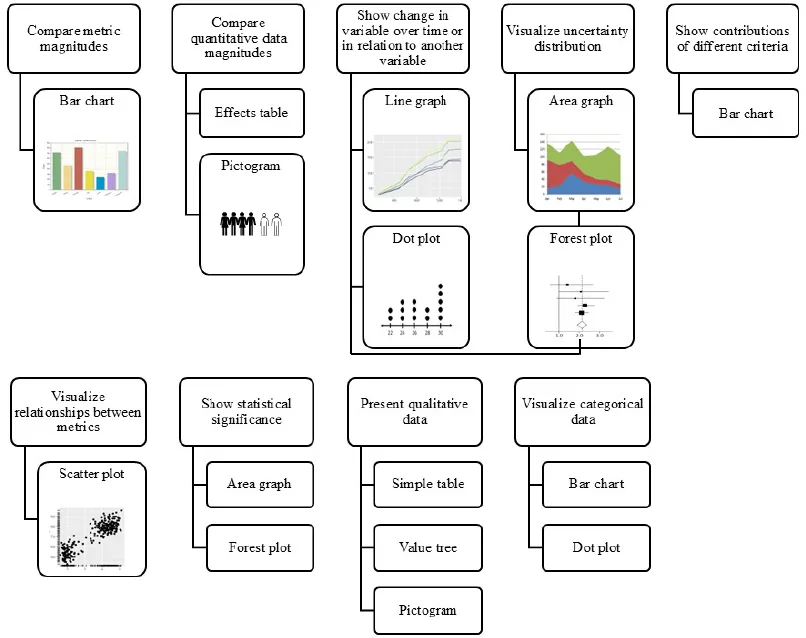
Synthesis of objectives and corresponding visual tools. Adapted from Hallgreen et al.^8^

## Final considerations

The choice of the appropriate visual tool depends on the type of information to be communicated, the target audience and the stage of the analysis. Value trees, effects and evidence tables, bar charts, scatter plots, line graphs, area and volume charts and pictograms, each tool having its own specific characteristics, play complementary roles in data presentation, helping to ensure that information is conveyed in an understandable way. A gradual approach can be adopted, starting with tools that offer an overview of benefits and risks and subsequently presenting the magnitudes of the effects. Furthermore, simultaneous use of different tools can be considered.

Careful application of these recommendations can contribute to transparency and consistency in data presentation, as well as to improving the effectiveness of benefit-risk assessment communication in health technology assessment processes.

## Data Availability

The data are available within the body of the manuscript.
