# Supplementary material for: Effective communication in benefit-risk assessment: the role of visual tools
Source: Epidemiol Serv Saude. 2026 Jun 24;35:e20251403. doi: 10.1590/S2237-96222026v35e20251403.en (PMC13686287; doi:10.1590/S2237-96222026v35e20251403.en)
Supplement: Translation

# Translation: Comunicação eficaz na avaliação benefício-risco: o papel das ferramentas visuais

**DOI:** 10.1590/S2237-96222026v35e20251403.pt

**Authors:** Ana Carolina Nonato, Erica Aranha Suzumura, Bruna de Oliveira Ascef, Aline Frossard Ribeiro Bortoluzzi, Fernando Henrique de Albuquerque Maia, Sidney Marcel Domingues, Beate Jahn, Uwe Siebert, Patrícia Coelho de Soárez

**Affiliations:** 1 Universidade de São Paulo, Faculdade de Medicina, São Paulo, SP, Brasil; 2 UMIT TIROL - University for Health Sciences and Technology, Institute of Public Health, Medical Decision Making and Health Technology Assessment, Hall in Tirol, Tirol, Áustria; 3 ONCOTYROL - Center for Personalized Cancer Medicine, Division of Health Technology Assessment, Innsbruck, Tirol, Áustria; 4 Harvard T.H. Chan School of Public Health, Center for Health Decision Science and Departments of Epidemiology and Health Policy & Management, Boston, MA, Estados Unidos; 5 Harvard Medical School, Mass General Brigham Center for Health Technology Assessment, Boston, MA, Estados Unidos

Ferramentas visuais desempenham um papel fundamental na avaliação benefício-risco, por facilitarem a comunicação de informações complexas e aprimorarem a compreensão entre as diferentes partes interessadas. Este artigo apresenta os principais tipos de ferramentas para apresentação de avaliação benefício-risco utilizadas no contexto da avaliação de tecnologias em saúde e discute aspectos importantes para sua seleção e sua aplicação adequada.

## Principais tipos e características das ferramentas visuais

Uma revisão de escopo ^1^ sobre diretrizes metodológicas e publicações que orientaram a condução e o relato de avaliação benefício-risco ao longo do ciclo de vida de tecnologias em saúde identificou, em 74 documentos, os tipos de ferramentas visuais mais frequentemente utilizadas. As tabelas de efeitos e de evidências foram as ferramentas mais empregadas (n=49; 59,0%), seguidas pelas árvores de valor (n=31; 41,0%), os gráficos de barras (n=28; 33,7%), os gráficos de pontos (n=28; 33,7%), os gráficos de linhas (n=26; 31,3%) e os gráficos de área (n=24; 28,9%). As categorias não eram mutuamente excludentes, de forma que era comum que uma mesma publicação utilizasse mais de um tipo de ferramenta visual.

Entre as diversas ferramentas visuais utilizadas na avaliação benefício-risco, a árvore de valor (Figura 1) e a tabela de efeitos (Tabela 1) destacam-se por seu papel estruturante na organização dos critérios e na consolidação das evidências quantitativas. Ainda que não estejam presentes em todas as avaliações, essas ferramentas são frequentemente utilizadas como base para a definição dos desfechos relevantes e para a síntese transparente das informações que fundamentam o julgamento benefício-risco.

**Figura 1:** Desfechos selecionados para representar benefícios e riscos do uso de natalizumabe em relação ao comparador. Adaptado de Nixon et al. ^7^

**Tabela 1:** Benefícios e riscos associados ao natalizumabe em comparação ao placebo, segundo os desfechos avaliados. Adaptado de Nixon et al. ^7^

Resultado	Desfecho	Natalizumabe Risco absoluto por 1 mil pacientes	Placebo Risco absoluto por 1 mil pacientes	Diferença de risco absoluto por 1 mil pacientes (IC95%)	
Benefícios					
Benefícios de conveniência	Conveniência	-	-	--	
Benefícios médicos	Recaída	280,00	450,00	-260,00 (-326,000; -195,0)	
	Progressão da deficiência	110,00	230,00	-120,00 (--)	
**Riscos**					
Infecção	Reativação de infecção viral grave por herpes	80,00	70,00	10,00 (-26,00; 45,00)	
	Leucoencefalopatia multifocal progressiva	2,00	0,00	2,00 (--)	
Toxicidade hepática	Elevação das transaminases	50,00	40,00	10,00 (-16,00; 38,00)	
Toxicidade reprodutiva	Anomalias congênitas	-	-	-	--
Distúrbios neurológicos	Convulsões	-	-	-	--
Outros	Reações à infusão/injeção	236,00	180,00	56,00 (6,00; 114,00)	
	Reações de hipersensibilidade	90,00	40,00	50,00 (20,00; 82,00)	
	Reações semelhantes às da gripe	399,00	400,00	-1,00 (-114,00; 114,00)	

**Legenda:** verde indica a diferença no risco absoluto de ocorrência do evento favorável ao natalizumabe (benefício ou redução de eventos adversos em relação ao comparador); rosa indica a diferença no risco absoluto de ocorrência do evento desfavorável em relação ao natalizumabe (aumento de eventos ou maior risco em comparação ao placebo).

A árvore de valor representa de forma clara e estruturada a relação entre os benefícios e os riscos considerados na avaliação benefício-risco (Figura 1). Sua elaboração ocorre tipicamente em reuniões de equipes multidisciplinares responsáveis pela avaliação benefício-risco, nas quais ela atua como instrumento de discussão e de registro dos elementos mais relevantes do processo avaliativo. A **árvore de valor** concentra-se nos eventos principais relacionados à avaliação benefício-risco e não tem como finalidade apresentar uma lista exaustiva de eventos, seja na dimensão dos benefícios ou dos riscos, mas sim destacar os benefícios e os riscos mais relevantes que um tomador de decisão considera ao escolher entre diferentes tecnologias ^2^.

Essa ferramenta pode ser usada em qualquer metodologia que envolva múltiplos desfechos, a fim de facilitar a compreensão da estrutura do problema. Geralmente, uma árvore de valor é elaborada nas etapas iniciais das estruturas analíticas (*frameworks*) PrOACT-URL ^3^ ou BRAT ^4^ e pode ser também empregada para orientar o processo de atribuição de pesos em métodos quantitativos, como a análise de decisão multicritérios ^5^. É fundamental que uma árvore de valor seja apresentada juntamente com informações sobre o contexto em que foi criada, pois, isoladamente, ela não fornece detalhes suficientes sobre as razões para a inclusão ou a exclusão de certos critérios. Para evitar interpretações incorretas ou vieses, é essencial explicitar as premissas e garantir transparência na seleção dos benefícios e dos riscos apresentados em uma árvore de valor, com incorporação, idealmente, da experiência clínica e da perspectiva dos pacientes. Além disso, uma vez que uma árvore de valor não fornece dados quantitativos, como medidas de associação ou de efeito, pode levar a interpretações equivocadas se não for adequadamente avaliada, já que não indica associações nem medidas de força das evidências entre os critérios apresentados ^5^^),(^^6^. Também é importante assegurar que os eventos representados em uma árvore de valor correspondam aos eventos identificados nos estudos clínicos utilizados como fontes de evidências para a avaliação benefício-risco.

A Tabela 1, uma tabela de efeitos, apresentou uma visão geral dos valores numéricos dos benefícios clínicos e dos riscos associados a determinada tecnologia. Os dados exibidos em uma tabela de efeitos são puramente descritivos, ou seja, não incorporam nenhuma análise formal das informações de preferência. Em alguns casos, os dados podem evidenciar de maneira clara que uma tecnologia em saúde apresenta um balanço benefício-risco favorável em relação ao(s) seu(s) comparador(es). Quando os dados de uma tabela de efeitos não indicam vantagem clara de nenhuma tecnologia alternativa, por exemplo, quando a tecnologia de interesse oferece não só benefícios adicionais, mas também riscos adicionais, pode-se considerar a modelagem quantitativa para a avaliação benefício-risco com o cálculo de medidas explícitas de compensação, como a razão dano-benefício incremental ^5^.

Para além da apresentação descritiva, uma tabela de efeitos pode integrar dados de análises formais de benefício-risco, com inclusão da atribuição de pesos aos desfechos e do cálculo explícito das contribuições de benefício-risco. Nessa abordagem, evidências quantitativas e preferências relativas aos desfechos são incorporadas de forma transparente e estruturada, como ilustrou a Tabela 2. A tabela de efeitos é amplamente utilizada na estrutura analítica PrOACT-URL e na análise de decisão multicritérios ^6^^)-(^^8^. Em sua elaboração, é importante buscar equilíbrio entre a quantidade de informações apresentadas e a facilidade de leitura, pois tabelas excessivamente densas podem aumentar a carga cognitiva do leitor e dificultar a extração das informações mais relevantes. 

**Tabela 2:** Diferenças de risco absoluto de ocorrência do evento por 1 mil pacientes e respectivos intervalos de confiança de 95% (IC95%) para benefícios e riscos associados ao natalizumabe versus placebo, segundo os desfechos avaliados, ponderados pelo método de análise de decisão multicritérios^a^. Adaptado de Nixon et al. ^7^

	Desfecho	Natalizumabe Risco absoluto por 1 mil pacientes	Placebo Risco absoluto por 1 mil pacientes	Diferença na probabilidade por 1 mil pacientes(IC95%)	Peso (%)	Benefício-risco
Benefícios						
Benefícios de conveniência	Conveniência	--	--	--	1,00	-0,30
Benefícios médicos	Recaída (número de recaídas)	467,00	1460,00	-993,00 (-1267,00; -756,00)	8,00	3,97
	Progressão da deficiência	113,00	230,00	-117,00 (-124,00; -110,00)	5,00	0,59
**Riscos**						
Infecção	Reativação de infecção viral grave por herpes	0,00	0,00	0,00 (-0,00; 6,30)	6,00	0,00
	Leucoencefalopatia multifocal progressiva	1,51	0,00	1,51 (0,00; 3,00)	54,00	-0,08
Toxicidade hepática	Elevação das transaminases	50,00	40,00	10,00 (-19,00; 36,00)	11,00	-0,11
Toxicidade reprodutiva	Anomalias congênitas	0,00	0,00	0,00 (-6,00; 3,00)	5,00	0,00
Distúrbios neurológicos	Convulsões	5,00	5,00	0,00 (-2,00; 12,00)	5,00	0,00
**Outros**	Reações à infusão/injeção	236,00	0,00	236,00 (202,00; 269,00)	3,00	-0,71
	Reações de hipersensibilidade	0,00	0,00	0,00 (-6,00; 3,00)	1,00	0,00
	Reações semelhantes às da gripe	399,00	400,00	-1,00 (-90,00; 86,00)	1,00	0,00
**Total**					100,00	3,36

^a^Esta tabela apresenta a avaliação estruturada de benefício-risco, com a comparação do natalizumabe ao placebo e a utilização do framework PrOACT-URL e do método de análise de decisão multicritérios. Os desfechos estão agrupados em domínios de benefício e de risco (nas primeiras colunas). Os riscos absolutos de ocorrência dos eventos por 1 mil pacientes para natalizumabe e placebo são apresentados, seguidos da diferença de risco absoluto com seus respectivos intervalos de confiança de 95% (IC95%). Cada desfecho recebeu um peso relativo definido na análise de decisão de multicritérios. A contribuição benefício-risco de cada critério foi calculada a partir da diferença de risco absoluto ponderada pelo respectivo peso. O resultado global de benefício-risco é apresentado na última linha como a soma ponderada de todos os critérios. Valores positivos indicam que os benefícios do natalizumabe superam os riscos em comparação com o placebo.

**Legenda:** A codificação por cores da coluna de diferença de risco absoluto indica a direção do efeito: verde indica desfechos favoráveis ao natalizumabe; vermelho indica desfechos favoráveis ao comparador.

A tabela de evidência é semelhante à tabela utilizada para a apresentação da certeza da evidência avaliada segundo os critérios propostos pelo método GRADE (Grading of Recommendations assessment, Development and Evaluation) ^(^^9^. Na avaliação benefício-risco, uma tabela de evidência deve incluir desfechos considerados importantes ou críticos, sejam benefícios ou riscos, e a qualidade da evidência deve ser avaliada e apresentada, com indicação do nível de certeza nas estimativas de efeitos e dos fatores que a influenciam, como o risco de viés, de inconsistência ou de imprecisão ^10^^),(^^11^.

O gráfico de barras pode ser empregado para comparar eficácia e segurança de diferentes tratamentos, ilustrar a incidência de eventos adversos ou demonstrar a distribuição dos resultados em diferentes grupos de pacientes. É importante ressaltar que, ao utilizar o gráfico de barras nesse contexto, deve-se ter cuidado para garantir que a escala e as proporções sejam representadas de forma precisa ^6^^)-(^^8^.

O gráfico de pontos é uma alternativa simplificada ao gráfico de barras e pode exibir uma variedade de medidas, como frequências, probabilidades, proporções e outros resultados por grupo. A principal vantagem dessa ferramenta é que ela apresenta apenas os pontos de dados cruciais, com eliminação dos elementos visuais desnecessários.

O gráfico de floresta (*forest plot*) é uma variação mais sofisticada do gráfico de pontos, com incorporação de fundamentos estatísticos mais robustos, e é particularmente útil para comunicar medidas sumárias, como diferenças entre riscos absolutos e razões de risco, com seus respectivos intervalos de confiança. Essa característica torna o gráfico de floresta especialmente valioso na comunicação da avaliação benefício-risco, pois permite uma avaliação visual rápida da magnitude e da precisão dos efeitos observados ^6^^)-(^^8^.

O gráfico de linhas comunica relações e mudanças em medidas ao longo de um intervalo de valores, o que permite a visualização de tendências, padrões e relações entre variáveis. É particularmente útil para evidenciar relações de dose-resposta (por exemplo, a frequência de eventos adversos em relação à dosagem de um medicamento) ou para ilustrar variações na eficácia de um tratamento ao longo do tempo ^6^^)-(^^8^.

O gráfico de área e de volume pode ser usado para comparar visualmente a prevalência de riscos e de benefícios entre tecnologias alternativas. Uma limitação significativa desses gráficos é a dificuldade humana em perceber com precisão áreas e volumes. Consequentemente, embora esse gráfico possa efetivamente ilustrar a existência de diferenças nos efeitos observados entre diferentes tecnologias em saúde, pode ser difícil determinar com exatidão a magnitude dessas diferenças ^6^^)-(^^8^.

Os pictogramas, por sua vez, desempenham um papel importante na transmissão de informações complexas de forma acessível para diversos públicos, como reguladores, pacientes e público geral, pois são compreendidos mais facilmente do que outros gráficos. Podem ser utilizados para representar visualmente a incidência de eventos adversos e benefícios de tratamentos ou outras tecnologias em saúde, com utilidade particular para comunicar probabilidades e proporções ^6^^)-(^^8^.

## Seleção da ferramenta visual adequada

A seleção da ferramenta visual adequada para a avaliação benefício-risco depende de vários fatores, como o tipo de informação a ser comunicada, o público-alvo e a fase da análise. Deve-se determinar a compatibilidade entre as imagens e o público-alvo e qual a principal mensagem a ser comunicada, com a consideração do nível de conhecimento necessário para a compreensão do gráfico ou da figura, e se há informações suficientes para que a mensagem pretendida seja comunicada e compreendida claramente ^6^^)-(^^8^.

Na fase de planejamento da avaliação, recomenda-se utilizar a árvore de valor para visualizar a estrutura hierárquica do problema decisório, além de preparar um modelo de tabela para representar os dados a serem coletados. Durante a coleta de evidências e a preparação dos dados, a tabela de efeitos deve ser preenchida, com destaque das informações disponíveis e ausentes. Para apresentação ao público em geral, sugere-se o uso de escalas de risco ou de pictogramas ^6^^)-(^^8^.

Na etapa de análise quantitativa, as preferências de valor das partes interessadas e as magnitudes de benefício-risco podem ser representadas por gráficos de barras, gráficos de pontos, gráficos de linha ou gráficos de floresta. Essas representações visuais promovem uma leitura precisa dos pontos, comparações locais e globais e facilitam o julgamento entre alternativas. Por fim, na fase de exploração, quando os resultados são verificados quanto à robustez devido a mudanças nos parâmetros e à incerteza estatística, recomenda-se o uso de um gráfico de distribuição, um diagrama de tornado ou um gráfico de fronteira para representar a distribuição ou a incerteza de uma medida ^6^^)-(^^8^. 

A Figura 2 sistematizou os objetivos e os principais tipos de ferramentas visuais a serem utilizados. Exemplos adicionais dessas ferramentas, desenvolvidas pelo projeto PROTECT BR ^6^, estão disponíveis para consulta em: https://imi-protect-eu.cc.ic.ac.uk/visualisations.html. 

**Figura 2:** Síntese dos objetivos e das ferramentas visuais correspondentes. Adaptado de Hallgreen et al.^8^

## Considerações finais

A escolha da ferramenta visual adequada depende do tipo de informações a serem comunicadas, do público-alvo e da fase da análise. Árvore de valor, tabelas de efeitos e de evidências, gráfico de barras, gráfico de pontos, gráfico de linhas, gráfico de área e de volume e pictogramas, cada ferramenta com suas especificidades, desempenham papéis complementares na apresentação dos dados, o que ajuda a garantir que as informações sejam transmitidas de maneira compreensível. Pode-se adotar uma abordagem gradual, com início por ferramentas que ofereçam uma visão geral dos benefícios e dos riscos e, posteriormente, com apresentação das magnitudes dos efeitos. Além disso, pode-se considerar o uso simultâneo de diferentes ferramentas.

A aplicação criteriosa dessas recomendações pode contribuir para a transparência e a consistência na apresentação dos dados, além de melhorar a eficácia na comunicação de avaliação benefício-risco nos processos de avaliação de tecnologias em saúde.

## Data Availability

Os dados estão disponíveis no corpo do manuscrito.
